# Metallic surface doping of metal halide perovskites

**DOI:** 10.1038/s41467-020-20110-6

**Published:** 2021-01-04

**Authors:** Yuze Lin, Yuchuan Shao, Jun Dai, Tao Li, Ye Liu, Xuezeng Dai, Xun Xiao, Yehao Deng, Alexei Gruverman, Xiao Cheng Zeng, Jinsong Huang

**Affiliations:** 1 Department of Applied Physical Sciences, University of North Carolina, Chapel Hill, NC 27599 USA; 2 Department of Chemistry, University of Nebraska–Lincoln, Lincoln, NE 68588 USA; 3 Department of Physics and Astronomy, University of Nebraska–Lincoln, Lincoln, NE 68588 USA; 4 Department of Mechanical and Materials Engineering, University of Nebraska-Lincoln, Lincoln, NE 68588 USA; 5 Department of Chemical & Biomolecular Engineering, University of Nebraska-Lincoln, Lincoln, NE 68688 USA

**Keywords:** Electronic devices, Electronic properties and materials

## Abstract

Intentional doping is the core of semiconductor technologies to tune electrical and optical properties of semiconductors for electronic devices, however, it has shown to be a grand challenge for halide perovskites. Here, we show that some metal ions, such as silver, strontium, cerium ions, which exist in the precursors of halide perovskites as impurities, can n-dope the surface of perovskites from being intrinsic to metallic. The low solubility of these ions in halide perovskite crystals excludes the metal impurities to perovskite surfaces, leaving the interior of perovskite crystals intrinsic. Computation shows these metal ions introduce many electronic states close to the conduction band minimum of perovskites and induce n-doping, which is in striking contrast to passivating ions such as potassium and rubidium ion. The discovery of metallic surface doping of perovskites enables new device and material designs that combine the intrinsic interior and heavily doped surface of perovskites.

## Abstract (web summary)

Intentional doping is important in semiconductors in order to tune the material property, yet the mechanism in metal halide perovskite is not well-understood. Here, the authors use silver, strontium, and cerium ions to showcase n-type doping on perovskite surface even up to metallic state.

## Introduction

Metal halide perovskite materials have been a research hotspot in the field of optoelectronics and new electronics, including solar cells^1–6^, photodetectors^7^, light-emitting diodes^8,9^, ionization radiation detection^10,11^, laser^12^, single-photon emitter^13^, spintronic devices^14^, etc. Excellent performance have been frequently observed owing to their exceptional intrinsic properties including long charge-carrier lifetime, high mobility, strong light absorption, tunable bandgap, long spin-relaxation lifetime, and efficient emission^15–21^. Among many electronic devices, doped semiconductors at designated location at certain doping concentration are needed for multiple purposes, such as ohmic contact formation, p-n junction formation, tuned resistivity, carrier recombination tuning, thermoelectric functionality, etc. However, intentional doping of metal halide perovskites remains to be a grand challenge. Most Pb-based perovskite materials are very good intrinsic semiconductors in their single crystalline form, whereas addition of elements with either more or less valence electrons does not obviously change their conductivity^22–25^. Various added metal ions, like potassium^26^ and rubidium ion^27^, have shown different functions in perovskite thin film and crystals including alloying, defect passivation, and/or accompanied tailoring of perovskite crystallization process^25^. Self-doping has been observed in Pb perovskites with non-stoichiometric compositions, however the doping concentration either has a very small tunable range or is not easily controlled^28–30^.

Here, we show that some metal ions such as silver, strontium, cerium ions, which exist in the precursors for metal halide perovskites as impurities, can n-dope the surface of perovskites from being intrinsic all the way upto metallic. We find these metal ion additives prefer to stay at perovskite surfaces because of their low solubility in metal halide perovskite crystals, leaving interior of perovskite crystals intrinsic. Computation shows that these metal ions introduce many electronic states close to the conduction band minimum of perovskites and induce n-doping, which is in striking contrast to passivating ions such as potassium^26^ and rubidium ion^27^.

## Results

### Surface doping of perovskites

**Fig. 1:**
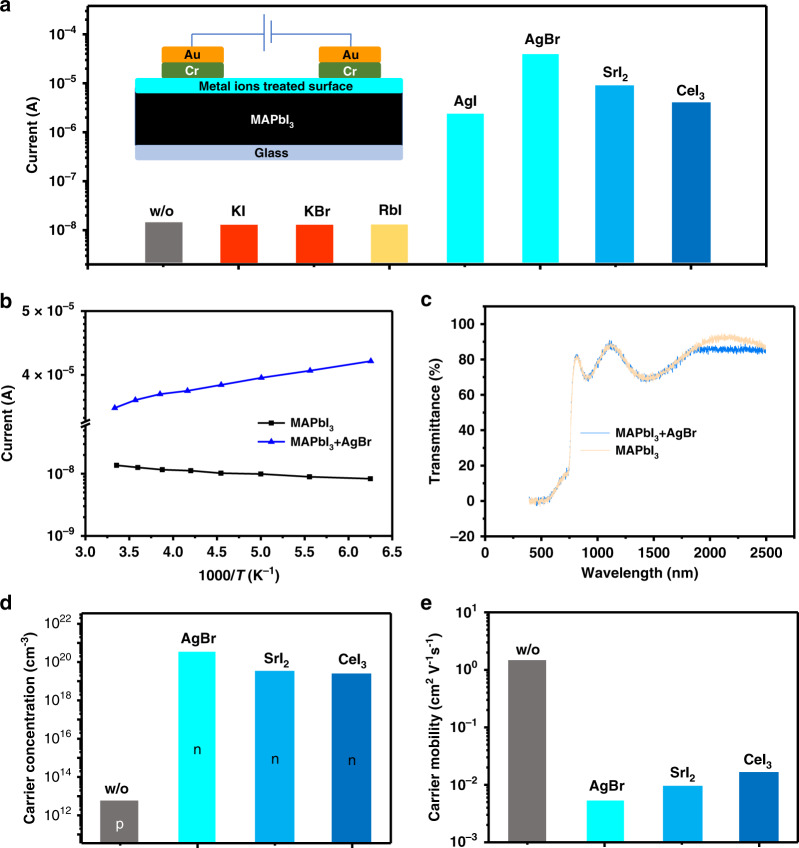
Surface doping of perovskites by some metal ions. **a** Dark current of lateral structure devices based on MAPbI_3_ thin films without and with treaded surface by different metal ions, and the inset shows schematic lateral device structure, in which the width of electrodes is 1 mm, channel length is 50 μm, and the applied bias is 20 V. **b** Dark current of lateral devices based on MAPbI_3_ thin films without and with AgBr surface treatment under different temperature, showing a transition from semiconducting to metallic properties after surface treatment. **c** Transmittance of the same MAPbI_3_ thin film before and after AgBr surface treatment. **d** Majority carrier type and concentration, and **e** mobility of MAPbI_3_ thin films with and without surface treated by AgBr, SrI_2_, and CeI_3_, calculated from Hall effect measurement and four-probe conductivity measurement.

Semiconductor to metal transition of perovskite surfaces was discovered in our measurement of the conduction change of halide perovskite thin films, which are covered with metal impurity ions. Here, CH_3_NH_3_PbI_3_ (MAPbI_3_) polycrystalline thin films (ca. 500 nm thick, 1–2 µm grain sizes) were first covered by silver, strontium, cerium halide powders, and the metal halide powders were removed after thermal annealing. After this simple treatment, we observed a huge increase of dark current of lateral devices based on MAPbI_3_ film by up to four orders of magnitude in lateral structure devices as illustrated in Fig. 1a. In striking contrast, the same treatment of MAPbI_3_ films using KI, KBr, or RbI does not obviously change perovskite device dark current, which also indicates the dark current increase comes from the effect of metal cations, rather than halide anions. The dark currents of MAPbI_3_ lateral devices further increase when AgBr were used for surface treatment to replace AgI (Fig. 1a). Furthermore, we used four-probe measurement of lateral structure devices to avoid possible impact of contact resistance to total current. The four-probe measurement results as shown in Supplementary Fig. 1 confirmed the huge enhancement of film conductivity upon doping. The dramatically increasing dark current indicates that the MAPbI_3_ surfaces are doped by these ions, and maybe already to become metallic states. To verify this, we measured temperature-dependent current of lateral devices based on MAPbI_3_ films without and with surface treatment by Ag^+^. In an intrinsic semiconductor such as halide perovskites, electronic conduction should increase with temperature (*T*) owing to increased carrier concentration if the mobility variation is relatively small. In contrast, metallic conductors show a decrease of conduction with increasing temperature because of increased carrier scattering induced by lattice^31^. As shown in Fig. 1b, the current of lateral device based on the pristine MAPbI_3_ increases at higher temperature. The device based on ion-treated MAPbI_3_ thin films show increased dark current at lower temperatures, as a hallmark metallic feature. Here we measured dark current at relatively low temperatures from 160 K to 300 K to exclude the contribution of ion migration to the total current^32^. Ion migration is completely frozen at low temperature such as 160 K, whereas the conductivity of perovskite with AgBr doping is still several orders of magnitude larger than that of pristine perovskites. This confirms the conduction is dominated by electronic contribution, rather than ionic migration of either perovskite or silver halide itself. In addition, the forward and reverse *I*^–^*V* scan curves of the lateral device based on AgBr-doped MAPbI_3_ showed negligible hysteresis (Supplementary Fig. 1c), which agrees with that the electronic contribution dominates the conduction, because the obvious ion migration should cause the current hysteresis. Another experiment also indicated the current increase is caused by doping perovskite, rather than the ionic conductivity of ion conductor like silver halides. To further confirm that AgBr can dope MAPbI_3_, we mixed 10 wt.% AgBr in MAPbI_3_ in solution, and make a mixed film. We measured the conductivity of MAPbI_3_ thin film with and without AgBr additive. As shown in Supplementary Fig. 2, the current of lateral device with large channel length (0.95 cm) based on MAPbI_3_ mixed with 10 wt.% AgBr was about four orders higher than that of the control device based on MAPbI_3_.

We also measured the transmittance of a MAPbI_3_ thin film before and after surface treatment by AgBr to probe the doping induced optical property change. Here the same MAPbI_3_ thin film was used to avoid the variation of composition or morphology. A decrease of transmittance in the wavelength range of 2000–2500 nm is observed for the MAPbI_3_ film with a Ag^+^-treated surface, as shown in Fig. 1c. It can be attributed to the enhanced plasma reflectivity of heavily doped MAPbI_3_. Here, the calculated carrier concentration of doped perovskite reaches 6.6 × 10^20^ cm^−3^ from the peak of the plasma reflection spectrum based on Drude model^33^. Such a high carrier concentration is consistent with the transition to metallic states after doping of MAPbI_3_. This indicates the carrier concentration of ion doped perovskite increased by 6–8 orders of magnitude compared with what were reported in the bulk of polycrystalline perovskite films^30^. We noted that the dark current in lateral devices only increased by 3–4 orders of magnitude, which can be explained by the surface doping effect, decreased charge-carrier mobility at high carrier concentration, as well as the non-perfect connectivity of the surface doped layer. Meanwhile, the Sr^2+^- and Ce^3+^-treated MAPbI_3_ thin film did not show obvious plasma peaks in transmittance spectra between 300 nm and 2500 nm, which indicates the carrier density of SrI_2_- and CeI_3_- treated MAPbI_3_ surface is much lower than AgBr-treated surface. To verify it, Hall effect measurement was conducted to investigate carrier density, doping type and charge-carrier mobility of the perovskite surfaces treated by metal halides, and the measurement results are summarized in Fig. 1d, e and Supplementary Table 1. Ag^+^, Sr^2+^, and Ce^3+^ ions treated MAPbI_3_ surfaces showed n-type behavior. In contrast, as-cast MAPbI_3_ thin film is weakly p-type with a hole concentration of 5.3 × 10^12^ cm^−3^. The calculated electron concentration of perovskite surfaces treated by AgBr, SrI_2_, and CeI_3_ is 3.2 × 10^20^, 3.6 × 10^19^, or 2.5 × 10^19^ cm^−3^, respectively. The calculated plasma reflection peak of Sr^2+^ and Ce^3+^ ions doped MAPbI_3_ is far beyond 2500 nm, in consistent with the measurement results. The electron mobilities are 5.3 × 10^−3^, 9.5 × 10^−3^, or 1.7 × 10^−2^ cm^2^ V^−1^s^−1^ for AgBr, SrI_2_, and CeI_3_-treated MAPbI_3_ surfaces, which is 2–3 orders of magnitude lower than that (1.4 cm^2^ V^−1^s^−1^) of hole mobility in pristine films and can be well explained by the additional carrier scatter centers caused by dopants.

**Fig. 2:**
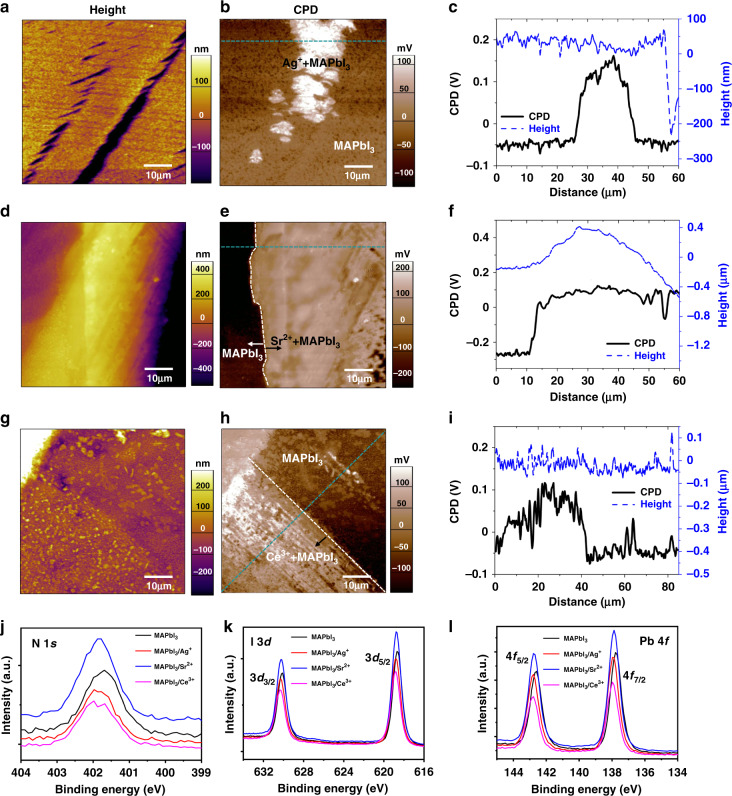
Doping type of perovskite surface doping. Height, CPD images and cross sectional curves of **a**–**c** AgI, **d**–**f** SrI_2_, or **g**–**i** CeI_3_ treated MAPbI_3_ single-crystal surface by KPFM measurement. **j**–**l** XPS scans of the N 1 *s*, I 3*d*, and Pb 4 *f* measured from the untreated MAPbI_3_ and MAPbI_3_ with surface treated by AgI, SrI_2_, and CeI_3_.

The work function change of MAPbI_3_ induced by doping was measured by Kelvin probe force microscopy (KPFM). We measured the area including the boundaries of the doped and undoped regions so that the change of work function among different regions can be quantified within one image. A clear boundary with large contact potential difference (CPD) is identified between pristine MAPbI_3_ crystal and metal halide treated regions, and the shape of the contact area depends on the size and shape of metal halide particles. The CPD in the measurements is defined as (Φ_tip_ ‒ Φ_sample_)/*e*. We used the same type of conductive tip (i.e., consistent Φ_tip_), thus CPD value should be directly related to the work function of the measured samples. The CPD line profile across the boundary shown in Fig. 2 reveals a higher CPD value, or lower work function, for metal halide treated area by *ca*. 160–260 meV, compared with the pristine surface region for MAPbI_3_. This result shows that all these metal ions n-dope the MAPbI_3_ surface. It should be noted here the untreated surface of MAPbI_3_ might be already partially self-doped by the volatilization of methylammonium iodide (MAI), and thus the change of surface work function by CPD cannot be directly used to calculate the doping concentration change of MAPbI_3_ crystals. The excess metal halides were cleanly removed and CPD change is not caused by metal halide itself, because the change of CPD by pure metal halide compared with MAPbI_3_ is opposite to the case of doping changed CPD. We deposited a ~5 nm thick AgI on a MAPbI_3_ surface in vacuum, and then conducted KPFM measurements at the edge between AgI covered perovskite surface and as-cast perovskite surface. Here we used pan paper to cover part of MAPbI_3_ surface, and thermally evaporated a AgI layer onto the uncovered part of MAPbI_3_. As shown in Supplementary Fig. 3, the CPD of AgI on perovskite surface is ~80 mV lower than that of as-cast MAPbI_3_ surface. In contrast, the CPDs of perovskite surface treated by metal halide doping are higher than that of MAPbI_3_. X-ray photoelectron spectroscopy (XPS) was used to investigate the surface chemistry of MAPbI_3_ thin film treated by Ag^+^, Sr^2+^, and Ce^3+^, respectively. There was no obvious metal halide residue detected within equipment sensitivity limit (Supplementary Fig. 4). All the peaks of N 1 *s*, Pb 4 *f*, and I 3*d* of the MAPbI_3_ surface treated by metal ions shifted to higher binding energy, as shown in Fig. 2j–l, indicating the moving up of the Fermi level, which is consistent with n-doping effect by the excess metal ions^34,35^.

### Distribution of metal ion dopants

**Fig. 3:**
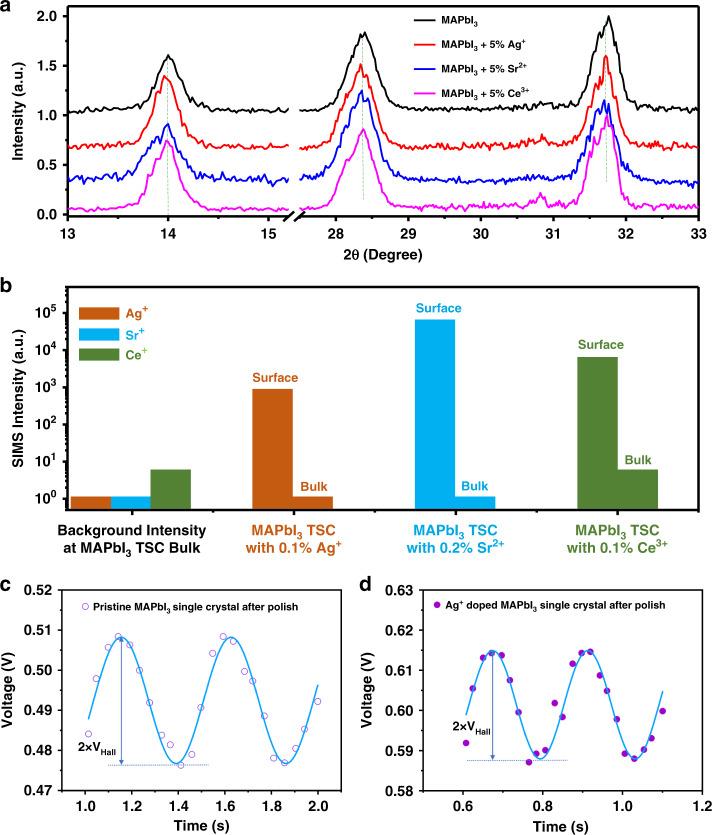
Distribution of metal ion dopants. **a** XRD curves of MAPbI_3_ powder without and with 5 wt.% Ag^+^, Sr^2+^ or Ce^3+^ additives. **b** TOF-SIMS intensity of Ag^+^, Sr^+^, and Ce^+^ on the surface and at the bulk (>800 nm depth) of MAPbI_3_ thin single crystals (TSCs) grown from precursor solution with 0.1% Ag^+^, 0.2% Sr^2+^, and 0.1% Ce^3+^ additives. Here the intensities of extrinsic metal ions within crystal bulk are at the level of background signals in MAPbI_3_ thin single-crystal grown from precursor solution without additive. **c**, **d** Hall effect measurements of MAPbI_3_ single crystals grown in precursor solutions **c** without and **d** with 0.1% Ag^+^ additives. We polished the surface of MAPbI_3_ singles crystals grown from precursor solution without and with metal ion additives, to avoid the impact of surface doping by the metal ions. The results show that the hole concentrations in the MAPbI_3_ single crystals grown in precursor solutions with and without Ag^+^ ions are 1.05 × 10^11^ cm^−3^ and 1.08 × 10^11^ cm^−3^, respectively. The almost same hole concentration indicates that the metal ions would not induce bulk doping.

One question rises naturally is whether these metal ions substitute Pb^2+^ or MA^+^ ions to cause a bulk doping within perovskite crystal lattices. Several experiment results can exclude this scenario. First, X-ray diffraction (XRD) characterization of both spun and blade-coated thin films (Supplementary Fig. 5) and polycrystalline powders (Fig. 3a) shows that no notable XRD peak shifts within equipment resolution limit has been observed after adding these ions into MAPbI_3_ at a concentration of 5 wt%. Second, we grew perovskite single crystals from perovskite precursor solutions with metal ion additives (0.1% Ag^+^, 0.2% Sr^2+^, 0.1% Ce^3+^ by weight ratio), and then measured the ion distribution using time-of-flight secondary ion mass spectrometer (ToF-SIMS). The ToF-SIMS intensities (Fig. 3b) of Ag^+^, Sr^+^, and Ce^+^ on the surface are much higher (3–5 orders) than those at the bulk of single crystals, and the intensities of extrinsic metal ions within crystal bulk are at the level of background signals in MAPbI_3_ thin single-crystal grown from precursor solution without additive. These results support that these extrinsic metal ions prefer to stay on the surfaces rather than incorporate into the crystals. Third, Hall effect measurement was conducted to characterize the bulk-doping concentration of these single crystals. The MAPbI_3_ crystal bulk is known to be weakly p-doped^15^. The almost invariant-free carrier concentrations and doping type in these single crystals grown from precursors with added metal ions also suggests that metal ion impurities do not incorporate into the perovskite lattice to cause bulk doping (Fig. 3c, d). Therefore, the observed dark current change of lateral structure devices merely comes from the surface doping of perovskites by the adsorption of metal ion impurities with perovskite surfaces. Ag^+^ and Sr^2+^ ions have been introduced into hybrid perovskite solar cells as additives to enhance the efficiency of solar cells^36–39^. It should also be noted the XRD peak shift of polycrystalline films cannot be simply explained by metal ion incorporation into perovskite lattice, unless the complexity of strain distribution in perovskite films could be well excluded^40^. Here we scraped the MAPbI_3_ powder with and without additives from the substrates for XRD study to exclude the impact of impact of strain caused by the substrates. No obvious change of XRD peaks is observed for the sample with 5% of metal additives, suggesting these metal ions do not easily incorporate MAPbI_3_, agreeing with TOF-SIMS study of single-crystal samples.

## Discussion

**Fig. 4:**
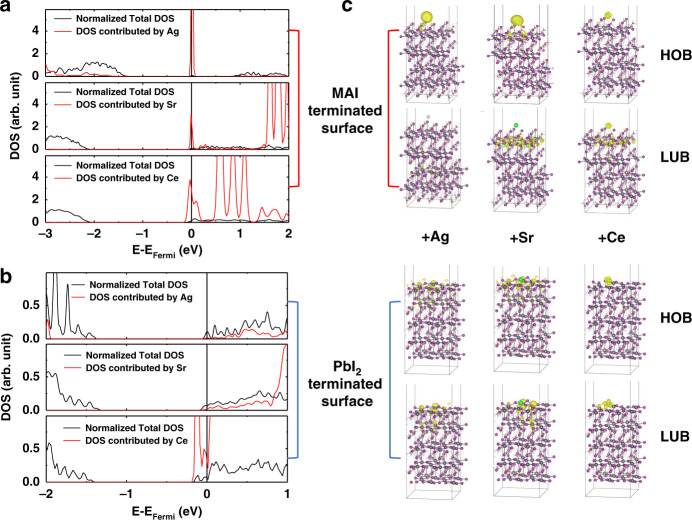
Modeling of surface doping mechanism. Computed normalized total and partial DOS of Ag, Sr, and Ce adsorbed on **a** MAI-terminated and **b** PbI_2_-terminated surfaces of MAPbI_3_. **c** The iso-surface plot of the charge density of the highest occupied band (HOB) and the lowest unoccupied band (LUB) of Ag, Sr, and Ce adsorbed on MAI-terminated and PbI_2_-terminated surfaces of MAPbI_3_. The purple, grey, silver, green and chartreuse spheres represent I, Pb, Ag, Sr and Ce atoms, respectively. The charge density distribution is highlighted in yellow.

To find out why the extrinsic metal ions can n-dope the surface of metal halide perovskites, we carried out first-principles computation within the framework of density functional theory (DFT). The surface doping is realized through the additive metal ions on the surface, therefore the simulation models composed of MAPbI_3_ surfaces and metal ions are natural representations of the surface doping from the structural perspective. Although the geometry of excess Ag, Sr, and Ce ions in metal halide perovskites might be more complex as treated with the surface adsorption model, the adsorption of metal atoms using surface models captures the essential interaction between metal atoms and halide perovskites, and provide insights into the engineering of surface band edges upon doping. The computation shows that, unlike ions such as K^+^ and Rb^+^ reported previously (Supplementary Fig. 6)^26,27^, Ag (Sr or Ce) clearly introduces some occupied states below the Fermi level in the doped samples, and the computed finite density of states (DOS) at the Fermi level can be observed for the Ag (Sr or Ce)-doped samples, suggesting the metallic state (Fig. 4). The highest occupied level of Ag, Sr, and Ce are close to the conduction band minimum (CBM) of MAI- or PbI_2_-terminated surface (Supplementary Fig. 7). It should be noted that the occupied orbitals of isolated metal atoms above or near the lowest unoccupied band of perovskite is the necessary but not the sufficient condition of n-doping perovskite surface, when surface metal atoms like K etc., strongly hybridize with surface I atoms on both MAI terminal and PbI_2_ terminal of perovskite. The normalized total DOS and the DOS contributed from Ag (Sr or Ce) are plotted in Fig. 4a, b. The computed charge density corresponding to the highest occupied and lowest unoccupied level of the Ag, Sr, and Ce adsorbed on the MAI-terminated and PbI_2_-terminated surfaces are also plotted in Fig. 4c, from which we can clearly see the contributions of Ag, Sr, and Ce states to the charge density of the highest occupied states near the Fermi level. The stronger doping effect of Ag^+^ doped perovskite surface with the counter ion from I^−^ to Br^−^ can be attributed to the relatively higher ionization degree of Ag^+^ in AgI to AgBr^41^.

In summary, we discovered the surface of metal halide perovskites can be doped in a controlled manner via certain metal ions with carrier concentration changed by eight orders of magnitude. This study also explains the difficulty for the bulk-doping of perovskites, because most metal ions cannot incorporate into the crystal structure of metal halide perovskite. The non-uniform distribution of metal ion dopants in perovskite polycrystalline films may strongly impact halide perovskite optoelectronics in many different ways. Heavily doping perovskites can potentially lead to new applications such as thermoelectric energy conversion, given these perovskites have known low thermal conductivity. Non-uniform doping in polycrystalline perovskite thin film with optimized doping ratios can form homojunctions between individual perovskite grains and their adjacent grain boundaries and/or surface, which can be expected to minimize charge recombination by separating photogenerated electrons and holes spatially into different transport channels, and facilitating the charge separation in low-dimensional perovskites in solar cells, as well as other optoelectronics, such as photodetectors, radiation detectors, and light-emitting diodes.

## Methods

### Materials

MAI were synthesized according to the methods reported in literature^42^. Other raw materials and solvents were purchased and were used without further purification.

### Fabrication of perovskite thin films

The spin-coated perovskite thin film was fabricated by the anti-solvent method, and the composition of MAPbI_3_ were used here. The 80 μL precursor solution (1.3 M) was spun onto substrate at 2000 rpm for 2 s and 4000 rpm for 20 s, the sample was quickly washed with 130 μL toluene at during spin coating. Subsequently, the sample was annealed at 70 °C for 10 min and 100 °C for 10 min. The perovskite precursor solution was dissolved in mixed solvent of *N*, *N*-dimethylformamide and dimethyl sulfoxide with the volume ratio of 9:1. The same procedure is used to fabricate the MAPbI_3_ thin film with AgBr additive, by using the perovskite precursor solution with 10 wt.% AgBr additive. The blending ratio in this work mean the weight ratio of metal halide/lead iodide in precursor solution. For blade-coating perovskite film, we bladed films at 160 °C from the same perovskite precursor solution used in spin-coated processing, and then thermal annealed them at 100 °C for 30 min.

### Dark current measurement of lateral structure devices

For lateral devices, OIHP thin films were spin-coated on glass substrates, and symmetrical electrodes were thermally deposited by using shadow mask. We used Cr (15 nm)/Au (25 nm) as electrodes, and two probe and four-probe geometry of electrode pattern were used. Unless stated otherwise, the electrode width is 1 mm and the channel length between electrodes is 50 μm for two probe devices and 100 μm for four-probe devices, respectively. Unless stated otherwise, we recorded the stable currents of devices at a bias of 20 V in two probe devices and I–V measurement of four-probe devices was from zero bias to high bias (forward scan) scan. The absence of obvious current hysteresis of our device is confirmed by changing current scanning directions (Supplementary Fig. 1c). We put metal halide powder on the surface of MAPbI_3_ thin films, and then annealed samples at 85 °C for 15 min. After that metal halide powders were blew off by using high-pressure nitrogen knife. The metal halide powders were purchased from Alfa Aesar, and used as received. The metal halide powders have sizes of submicrometer to hundreds of micrometers, and thus likely have point contacts with the perovskite surfaces. Thermal annealing at 85 °C would drive the ion diffusion so as to increase the coverage of ions on the perovskite surface. The measurements were performed in a Lakeshore Probe Station at dark condition, and the samples were placed on a metal plate with its temperature being controlled by a heater and injected liquid N_2_. The temperature-dependent conductivity was measured in vacuum (*ca*. 10^−5^ torr).

### Transmittance measurement

Transmittance of the same MAPbI_3_ thin film before and after AgBr surface treatment was recorded using a PerkinElmer Lambda 1050 UV/VIS/NIR spectrometer. The carrier concentration *n* is calculated by^33^

1 $$n = \frac{{\omega _p^2m^ \ast \varepsilon _0\varepsilon _r}}{{e^2}}$$

where *ε*_0_ and *ε*_r_ is vacuum dielectric constant and relative permittivity, respectively. m* is charge-carrier effective mass, and *e* is elementary charge. *ω*_p_ is plasma frequency given by

2 $$\omega _p = \frac{{2\pi c}}{\lambda }$$

where *c* and *λ* is speed of light and wavelength, respectively. In perovskite thin film with AgBr-treated surface, *λ* is ~2250 nm, and thus *ω*_p_ is ~8.37 × 10^14^ Hz.

### Single-crystal growth

We followed the same single-crystal growth method with our previous published method of hydrophobic interface-confined crystal growth method^43^. PbI_2_ (7.376 g) and MAI (2.544 g) were added into 10 mL γ-butyrolactone solvent to obtain MAPbI_3_ precursor solution. Then AgI, SrI_2_, CeI_3_ were blended into MAPbI_3_ precursor solutions at weight ratios of 0.1%, 0.2%, or 0.1%, respectively. The solutions were dropped between two PTAA-covered substrates, and thin MAPbI_3_ single crystals with thickness of about 50 µm were grown by increasing temperature.

### XRD measurement

XRD measurements were performed with a Bruker-AXS D8 Discover Diffractometer. Bruker-AXS D8 Discover Diffractometer is configured in parallel beam geometry with Cu Ka radiation. It should be noted we used the scraped powder from the substrates for XRD measurement to exclude the impact of strain effect on the lattice constant^40^. We have shown that thermal annealing could induce strain in perovskite films grown on ITO substrates, while scraped powders are strain free^40^. The small amount of power gave wide XRD peaks. As shown in Supplementary Fig. 5, the full width at half maximums for the XRD peaks of perovskite thin films deposited by both spin coating and blade-coating process are comparable to those of perovskite single crystals in literature^24^. No notable XRD peak shift was observed within its resolution limit after adding 5% excess metal ions into perovskite thin films. In addition, the blade-coated perovskite film with 5% Sr^2+^ showed obvious orientation change relative to the pure MAPbI_3_ film.

### ToF-SIMS measurement

ToF-SIMS analyses were conducted using a TOF-SIMS V (ION TOF, Inc. Chestnut Ridge, NY) instrument equipped with a Bi_*n*_^m+^ (*n* = 1–5, *m* =  1, 2) liquid metal ion gun, Cs^+^ sputtering gun and electron flood gun for charge compensation. Both the Bi and Cs ion columns are oriented at 45° with respect to the sample surface normal. The instrument vacuum system consists of a load lock for rapid sample loading and an analysis chamber, separated by the gate valve. The analysis chamber pressure was maintained below 5.0 × 10^−9^ mbar. For depth profiles acquired in this study, 3 keV Cs^+^ with 18 nA current was used to create a 120 µm × 120 µm area, and the middle 30 µm × 30 µm area was analyzed using 0.4 pA Bi^3+^ primary ion beam. The analysis was done with the non-interlaced mode with three cycles of analysis and one cycle of sputtering to improve the detection limit. The positive secondary ion mass spectra were calibrated using NH_4_^+^, Pb^+^, PbI^+^, CsPb^+^, and CsPbI^+^.

### XPS measurement

XPS data were acquired on a Kratos Axis Ultra DLD spectrometer with a monochromatic Al Kα source and a base pressure of ca. 5 × 10^−9^ torr. Survey and high-resolution scans were taken with pass energies of 80 eV and 20 eV, respectively. All data was corrected to the C 1 *s* peak at 284.6 eV. MAPbI_3_ thin films without and with silver, strontium, and cerium iodide, were deposited on ITO substrates.

### Hall effect measurement

The details of the Hall effect measurement have been reported in previous publication^29^. We polished the surface of MAPbI_3_ singles crystals grown from precursor solution without and with metal ion additives, to avoid the impact of surface doping by the metal ions. The MAPbI_3_ thin films with ~500 nm thickness was used in Hall effect measurement. The detailed parameters are shown in Supplementary Table 1, and one typical 5 nm depth surface is adopted for MAPbI_3_ thin films with surface treated by metal halide. Hall measurements were conducted in air. The mobility is calculated by conductivity from four-probe measurement and carrier concentration from Hall effect. The carrier concentration *n* is determined by

3 $$n = \frac{{{\mathrm{I}}_{\mathrm{x}}{\mathrm{B}}_{\mathrm{z}}}}{{V_{\mathrm{H}}te}}$$

where **I**_x_ and **B**_z_ is applied constant current and magnetic field, respectively. *V*_H_ is measured Hall voltage. *t* is the thickness of the sample and *e* is elementary charge.

### KPFM measurement

To prepare the single-crystal sample for KPFM study, we deposited the metal iodide particles onto certain area of pure MAPbI_3_ thin single crystals on ITO/PTAA substrates, followed by thermal annealing at 80 °C for 10 min. Metal halide powders with sizes of tens micrometers for AgI, hundreds micrometers for SrI_2_, and less than micrometers for CeI_3_ on the surface of MAPbI_3_ crystals. The shape of contact area depends on the size and shape of metal halide particles. Before the KPFM measurements, the samples with particles on the surface were examined under the build-in optical microscope of atomic force microscopy (AFM) to locate the region with the edge of untreated and treated area. The measurement area was chosen by position the AFM tip at the edge of particle on samples where the position is memorized in the system. Then the AFM tip was lifted up, and the particles on perovskite surface were blown away by the nitrogen knife, leaving the area with edge of undoped and doped MAPbI_3_ surface for KPFM measurements. Finally, the AFM tip was engaged back to the original position. KPFM is an AFM-based surface imaging technique to acquire work function or surface potential of a material. In this work, the KPFM measurements were performed using a commercial AFM (MFP3D-BIO, Asylum Research, USA) and Pt/Ir coated silicon probes (PPP-EFM, Nanosensors, Switzerland). The standard 2-pass KPFM technique was employed. The first pass acquired the morphology information, whereas during the second pass, tip was lifted ~30 nm above the sample morphology based on the first pass and the surface potential or CPD was acquired. The CPD in our measurements is defined as (Φ_tip_ ‒ Φ_sample_)/*e*. Meanwhile, 1 V DC and 2 V AC biases were supplied to the conductive probe. The CPD value was measured as the DC bias that nullify the first resonance component of the electrostatic force between tip and sample surface. The observed CPD is a relative potential difference with respect to the conductive probe. All measurements were conducted in dry N_2_ to prevent sample degradation. During the measurements, the good quality of topography tracking (perfect matching of trace and retrace curves) was first ensured for each KPFM measurement via adjusting the scan rate and set point to minimize the topographic crosstalk. Second, the lift height was always maintained at about 30 nm above the surface for each measurement. KPFM measurements need both AC and DC inputs, where AC bias is for generating the harmonic electrostatic force while DC bias is supplied to nullify the 1st harmonic term, i.e., the CPD value. The 1 V DC potential is the initial set value send to the circuit. It is required by the equipment that the tip potential is more positive than the sample potential to initiate the scan. When the scan starts, the feedback loop is triggered and the CPD value will be updated and stored in time as the DC input. The CPD images were flattened by the zero order flatten process, and an offset was automatic subtracted during flatten process.

### Computation

First-principles computation was carried out in the framework of DFT as implemented in the VASP 5.4 program. The generalized gradient approximation in the form of Perdew–Burke–Ernzerhof was used for the exchange–correlation functional. The ion–electron interaction was treated with the projector-augmented wave method. Grimme’s DFT-D3 dispersion correction was adopted to describe the long-range van der Waals interactions. The structures of the metal-doped surfaces were obtained through three steps. First, we optimized the structure of bulk tetragonal MAPbI_3_, for which both the lattice and atomic positions were allowed to relax until the force on each atom was smaller than 0.02 eV/Å. Second, we cleaved the PbI_2_-terminated and MAI-terminated symmetric (001) slabs from the optimized MAPbI_3_ tetragonal structure, respectively, each has a super cell of 2 × 2 and nine layers of MAI and PbI_2_ in total. About 30 Å vacuum was added on top of the slab surface so that the interaction between the adjacent slabs can be neglected. We then re-optimized these cleaved slabs. In this step, the lattice constants were fixed while all the atomic positions were allowed to relax until the force on each atom was smaller than 0.02 eV/Å. Third, we added metal atoms on these optimized slabs. The initial distance between metal atoms and surface of the slab along the *z* direction (normal to the surface of the slab) was set to 4 Å to avoid the pre-constrained bonding between metal atoms and surface. These metal-doped slabs were then re-optimized during, which the lattice constants were fixed while all the atomic positions were allowed to relax until the force on each atom was smaller than 0.02 eV/Å in this step. The re-optimized metal-doped surface structures were undertaken for the electronic structure calculations, for which a 5 × 5 Monkhorst–Pack grid was used for all DOS calculations.

## Supplementary information

Supplementary Information

## Data Availability

The data that support the findings of this study are available from the corresponding author upon reasonable request.
