# Supplementary material for: Metallic surface doping of metal halide perovskites
Source: Nat Commun. 2021 Jan 4;12:7. doi: 10.1038/s41467-020-20110-6 (PMC7782511; doi:10.1038/s41467-020-20110-6)
Supplement: Supplementary file 1 — Supplementary Information [file 41467_2020_20110_MOESM1_ESM.pdf]

Supplementary Information

Metallic Surface Doping of Metal Halide Perovskites

Yuze Lin[1], Yuchuan Shao[1], Jun Dai[2], Tao Li[3], Ye Liu[1,4], Xuezeng Dai[1], Xun Xiao[1], Yehao Deng[1], Alexei Gruverman[3], Xiao Cheng Zeng[2,3,4,5], Jinsong Huang[1,4]*

[1]Department of Applied Physical Sciences, University of North Carolina, Chapel Hill, NC 27599, USA. *Email: jhuang@unc.edu

[2]Department of Chemistry, University of Nebraska–Lincoln, Lincoln, Nebraska 68588, USA

[3]Department of Physics and Astronomy, University of Nebraska–Lincoln, Lincoln, Nebraska 68588, USA

[4]Department of Mechanical and Materials Engineering, University of Nebraska-Lincoln, Lincoln, Nebraska 68588, USA

[5]Department of Chemical & Biomolecular Engineering, University of Nebraska-Lincoln, Lincoln, Nebraska 68588, USA

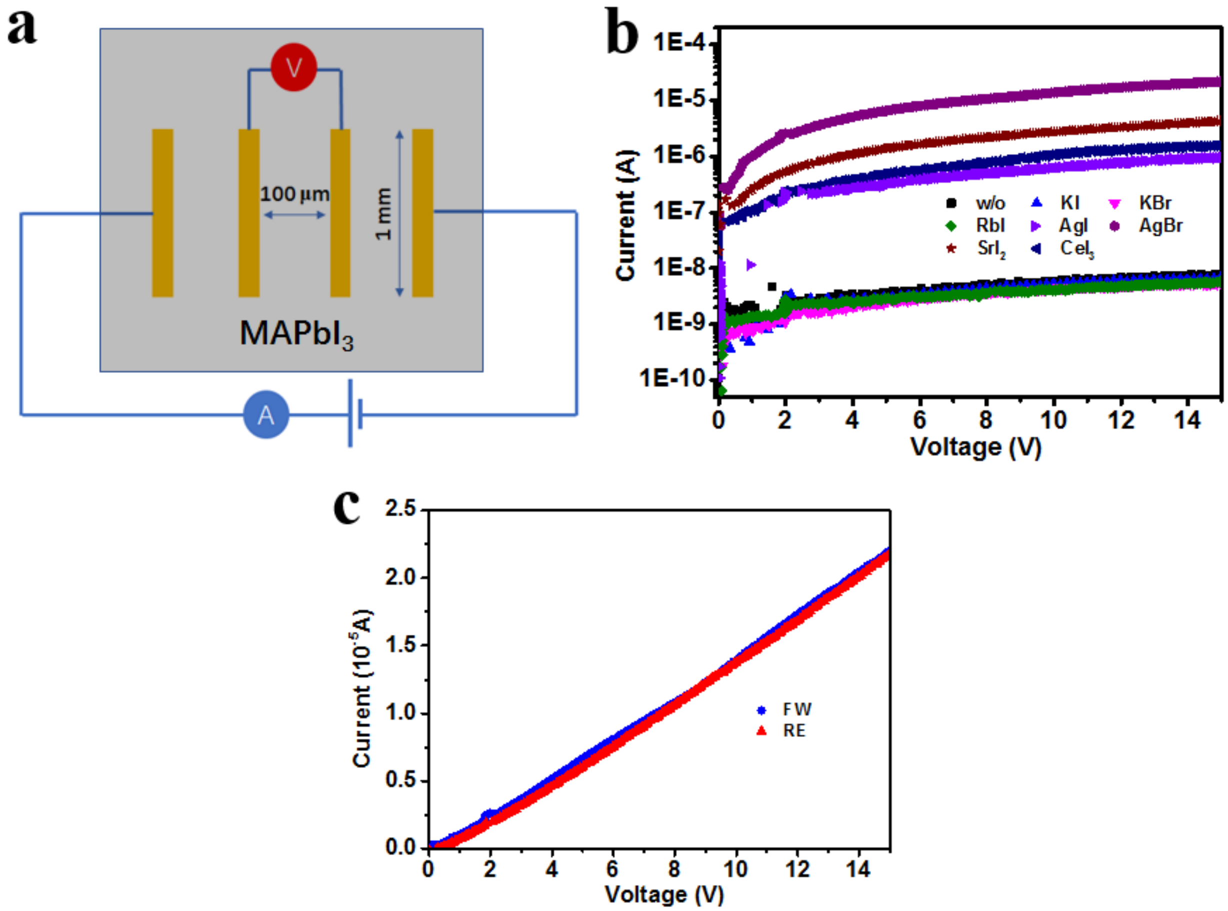

Supplementary Fig. 1. Dark current of lateral devices based on perovskite thin film with and without surface treatments. (**a**) The scheme of the device geometry of four probe lateral devices and (**b**) *I-V* curves of lateral devices based on $MAPbI_3$ with surface treated by different metal halide. (**c**) *I-V* curves of four probe lateral devices based on $MAPbI_3$ with surface treated by AgBr from forward (FW, zero bias to high bias) and reverse scan (RE, high bias to zero bias).

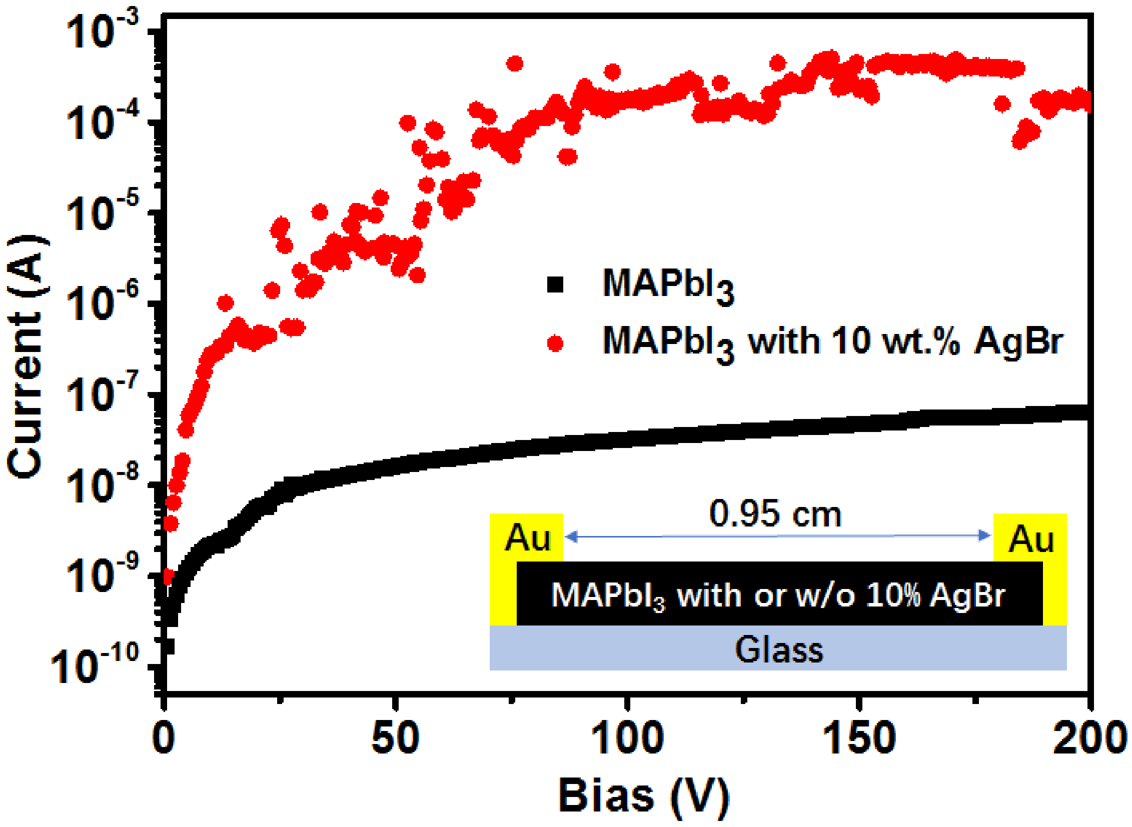

Supplementary Fig. 2. Dark current of lateral devices based on perovskite thin film with and without additive. *I-V* curves of lateral devices based on $MAPbI_3$ (~500 nm thickness) with or without 10 wt.% AgBr additive. The scan direction is forward scan (zero bias to high bias). The inset is the scheme of the device geometry of lateral devices, and the width of Au electrodes are 1.5 cm.

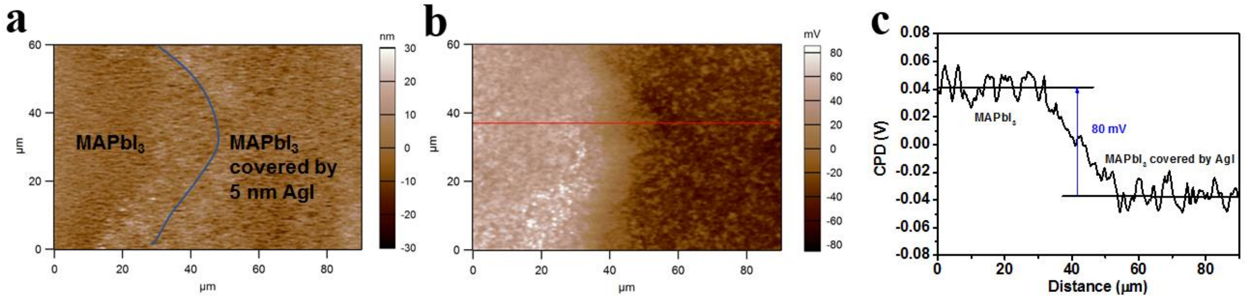

Supplementary Fig. 3. Morphology and potential of perovskite surface with and without AgI coverage. (**a**) Height and (**b**) CPD images (60 μm × 90 μm) of $MAPbI_3$ and $MAPbI_3$ covered by 5 nm AgI layer and (**c**) CPD cross-sectional curve.

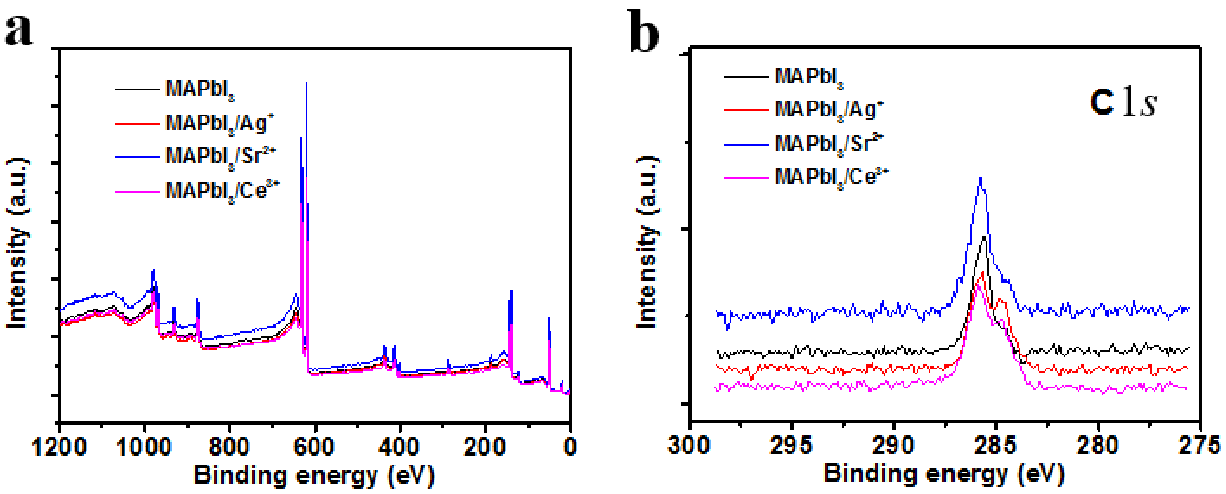

Supplementary Fig. 4. XPS of perovskite thin film with and without surface treatment. (**a**) XPS survey and (**b**) XPS scans of the C 1s measured from the untreated $MAPbI_3$ and $MAPbI_3$ with surface treated by AgI, SrI_2 and CeI_3.

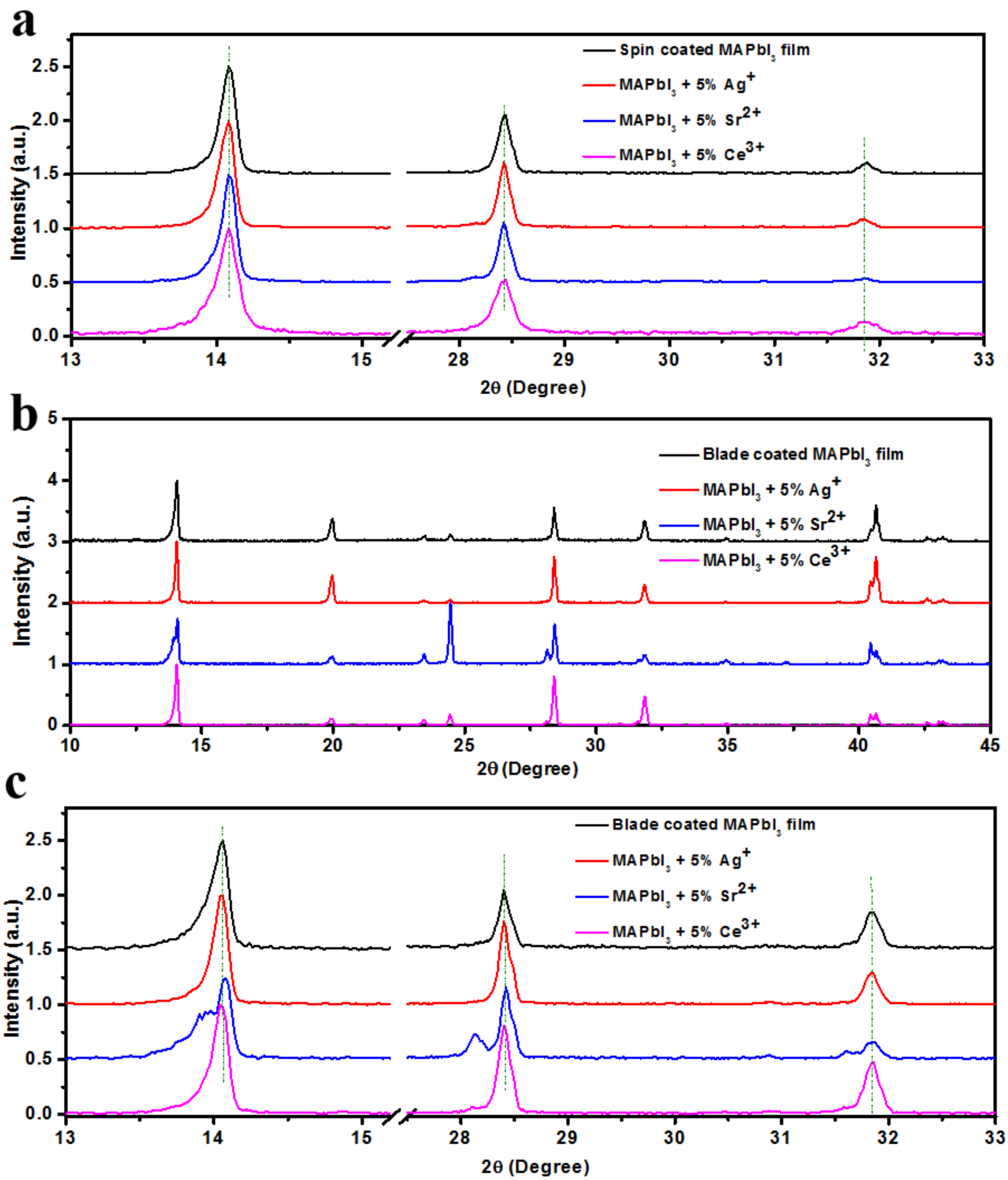

Supplementary Fig. 5. Distribution of metal ion dopants in spin- and blade-coated perovskite films. XRD curves of $MAPbI_3$ without and with 5 wt.% Ag^+, Sr^{2+} or Ce^{3+} additives: (**a**) spin coated films, (**b**-**c**) blade coated films.

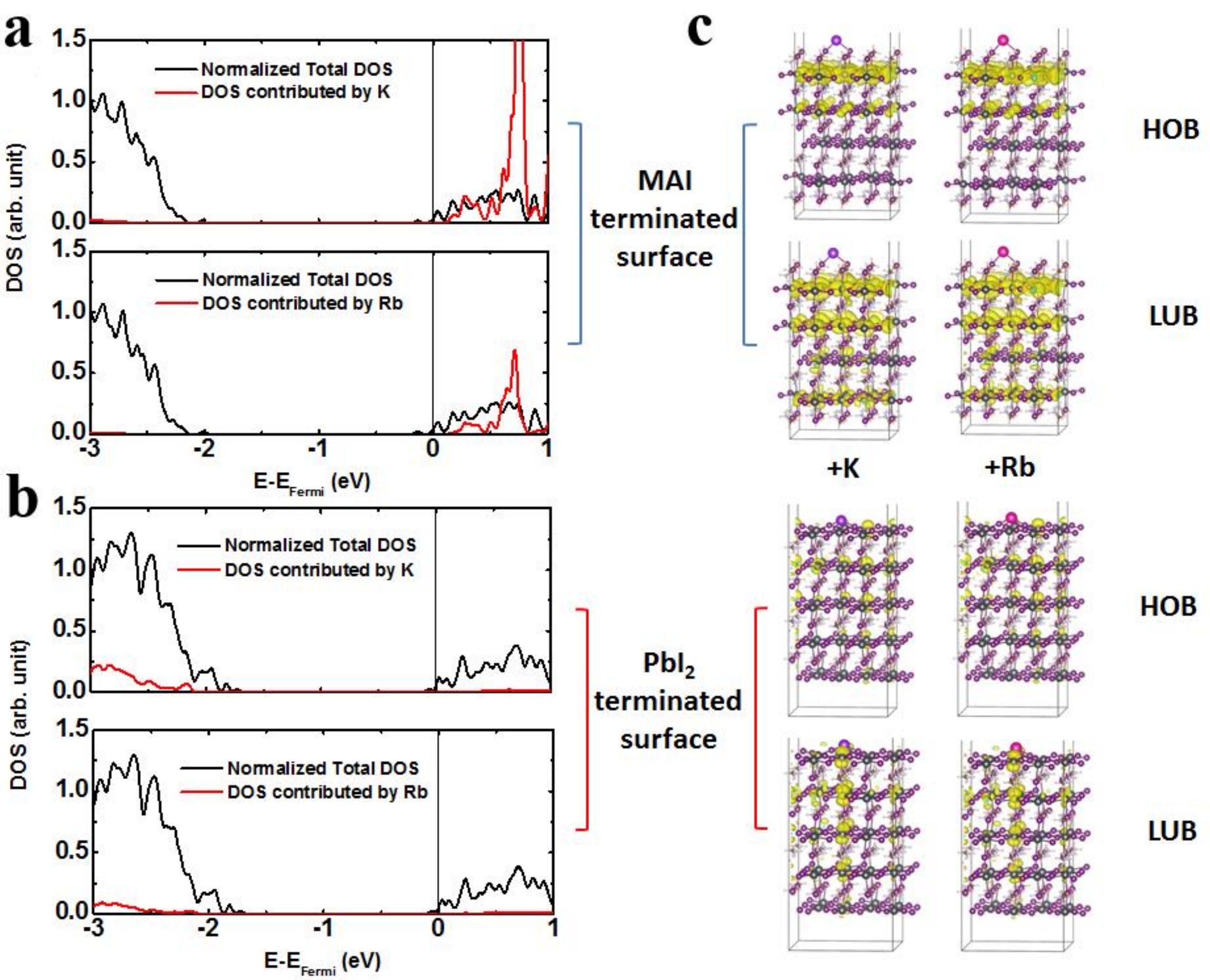

Supplementary Fig. 6. Modeling of K and Rb on the perovskite surface. Computed normalized total and partial density of states (DOS) of K and Rb adsorbed on (**a**) MAI-terminated and (**b**) PbI_2-terminated surfaces of $MAPbI_3$. (**c**) The iso-surface plot of the charge density of the highest occupied band (HOB) and the lowest unoccupied band (LUB) of K and Rb adsorbed on MAI-terminated and PbI_2-terminated surfaces of $MAPbI_3$.

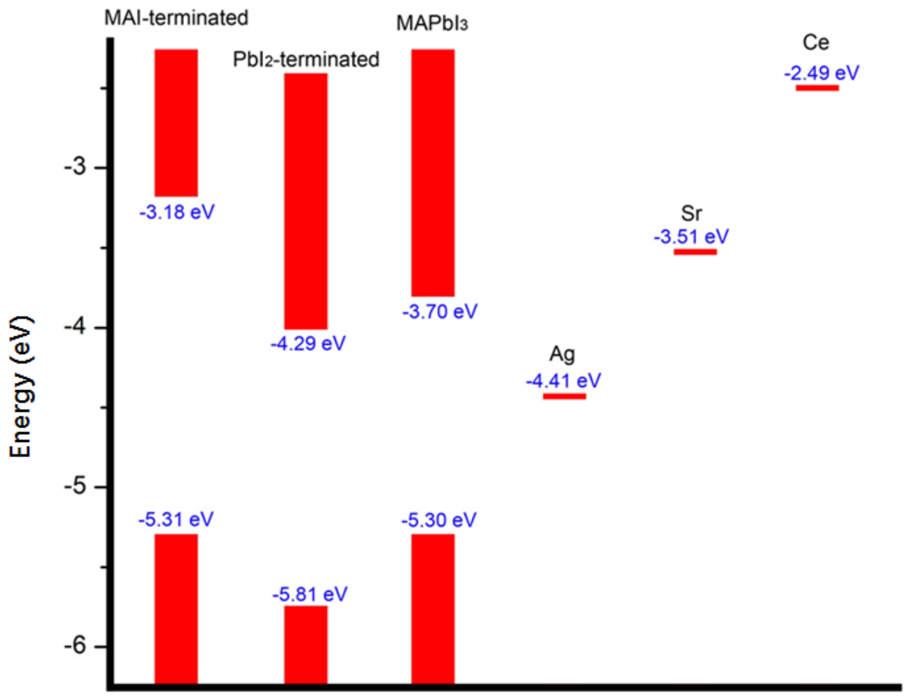

Supplementary Fig. 7. Energy levels. Energy levels of $MAPbI_3$, MAI (PbI_2) terminal surfaces, atomic Ag, Sr, and Ce.

Supplementary Table 1. The detailed parameters of the Hall effect measurement of $MAPbI_3$ thin film without and with surface treated by metal halide.

	$\mathbf{I}_x$	$\mathbf{B}_z$	V_H
$MAPbI_3$	0.42 nA	2T	-2 mV
$MAPbI_3$ treated by AgBr	50 μA	2T	0.4 mV
$MAPbI_3$ treated by SrI_2	10 μA	2T	0.7 mV
$MAPbI_3$ treated by CeI_3	10 μA	2T	1 mV
